# Chaos and Hyperchaos in a Model of Ribosome Autocatalytic Synthesis

**DOI:** 10.1038/srep38870

**Published:** 2016-12-12

**Authors:** Vitaly A. Likhoshvai, Vladislav V. Kogai, Stanislav I. Fadeev, Tamara M. Khlebodarova

**Affiliations:** 1 Institute of Cytology and Genetics, Siberian Branch, Russian Academy of Sciences, Novosibirsk, 630090 Russia; 2 Novosibirsk State University, Novosibirsk, 630090 Russia; 3 Sobolev Institute of Mathematics, Siberian Branch, Russian Academy of Sciences, Novosibirsk, 630090 Russia

## Abstract

Any vital activities of the cell are based on the ribosomes, which not only provide the basic machinery for the synthesis of all proteins necessary for cell functioning during growth and division, but for biogenesis itself. From this point of view, ribosomes are self-replicating and autocatalytic structures. In current work we present an elementary model in which the autocatalytic synthesis of ribosomal RNA and proteins, as well as enzymes ensuring their degradation are described with two monotonically increasing functions. For certain parameter values, the model, consisting of one differential equation with delayed argument, demonstrates both stationary and oscillatory dynamics of the ribosomal protein synthesis, which can be chaotic and hyperchaotic dependent on the value of the delayed argument. The biological interpretation of the modeling results and parameter estimation suggest the feasibility of chaotic dynamics in molecular genetic systems of eukaryotes, which depends only on the internal characteristics of functioning of the translation system.

Ribosomes are key structural and functional elements of any cell. They not only provide the basic machinery for the synthesis of all proteins necessary for cell functioning during growth and division, but for biogenesis itself. For the last several decades, various control aspects of ribosome synthesis, assembly and degradation have been studied intensively in both prokaryotes and eukaryotes (see review^1^,^2^,^3^,^4^,^5^,^6^,^7^,^8^,^9^,^10^,^11^). However, the dynamic aspects of functioning of this “protein factory” within a single cell cycle are still not clear.

From a biological point of view, ribosomes are self-replicating structures because they provide and control all stages of their own biogenesis. By participating in the synthesis of RNA polymerases, ribosomes assure the transcription of ribosomal and messenger RNAs and the subsequent translation of all structural and regulatory proteins that control maturation, assembly, functioning, recycling, degradation and synthesis of ribosomes *de novo*. That is why, reproduction of ribosomes is an autocatalytic process which at the molecular genetic level represents a gene network in which synthesis and degradation of rRNA, as well as ribosomal proteins and their regulators, are controlled by feedback mechanisms.

Theoretical studies of hypothetical gene networks and analysis of mathematical models of natural gene network have shown that, under certain conditions, processes controlled by a feedback mechanism exhibit chaotic dynamics^12^,^13^,^14^,^15^,^16^,^17^,^18^,^19^.

We have previously studied the role of retardation in generating chaos in genetic networks and have demonstrated that retardation existence (in an explicit or implicit form) is a prerequisite for chaos generation^17^, although quantitative estimates of retardation values are not known.

From the retardation point of view, transcription/translation systems in eukaryotic and prokaryotic organisms are contrasting genetic systems, yet main stages of their course are identical. The reason for this is the fact that prokaryotic transcription/translation processes are conjugated, that is, proceed almost simultaneously, while in eukaryotes they are separated into different compartments: transcription occurs in the nucleus and translation – in the cytoplasm (Fig. 1).

Even in a simplified form demonstrated in Fig. 1, the complexity and duration of the eukaryotic ribosome reproduction, accompanied by multiple transport processes, compared to prokaryotic ribosome reproduction, are clearly visible. In this sense, one can say that retardation is a natural phenomenon in eukaryotic gene networks. Perhaps that is why we usually observe chaotic dynamics in mathematical models of natural eukaryotic gene networks^14^,^18^,^20^,^21^,^22^,^23^,^24^,^25^,^26^.

However, it would be incorrect to *a priori* deny the existence of the retardation phenomenon in the gene networks of prokaryotic organisms. Gene networks of high dimensionality, controlled through complex nonlinear interactions, for which the formation of a chaotic dynamics was theoretically shown^27^, are characteristic of prokaryotes, likewise of eukaryotes.

From this point of view, the fundamental cellular processes common to all living organisms, including the biogenesis of ribosomes, which have chaos generating factors in their structural and functional organization, but demonstrate different dynamic characteristics in different organisms, are of particular interest.

In this paper, we present a theoretical analysis of the functioning dynamics using a model of a simple genetic system of ribosome biogenesis, which consists of two subsystems that describe positive autocatalytic synthesis of ribosomes and ribosome-degrading enzymes depending on the value of delay in the absence of any regulatory interactions.

We demonstrate that the model of ribosome biogenesis, in which the processes of ribosome synthesis and degradation are described with two monotonically increasing functions display chaotic and hyperchaotic dynamics of ribosome synthesis for certain parameter values. Previously, the presence of chaotic dynamics has been demonstrated only for systems described with unimodal and monotonically decreasing functions^15^,^17^,^18^,^19^,^28^.

## Model

The model describes a simple gene network of ribosome autocatalytic synthesis, which in general is the same in eukaryotic and prokaryotic organisms. By participating in the synthesis of RNA polymerases, ribosomes assure the transcription of ribosomal and messenger RNAs and the subsequent translation of regulatory proteins that control maturation, assembly and functioning of ribosomes. In this sense, ribosomes synthesize themselves. However, because RNA polymerase II synthesizes all kinds of messenger RNAs required for the cell functioning, it as well synthesizes the mRNA of protein-degrading enzymes (proteases) which have either lost their functionality, or the need for their presence in a cell has disappeared due to the changes in external conditions. By synthesizing these proteins, ribosomes enhance the process of their own degradation.

Thus, the model describes positive processes of ribosome self-synthesis and ribosomal protease synthesis, the functional features of which lead to the negative ribosome number regulation. The genetic circuit corresponding to this system is depicted in Figure SI1.2.

The process of ribosome self-synthesis and ribosomal protease synthesis is described in the model with one equation with two monotonically increasing functions and one retarded argument. In this case, the retarded argument reflects the time taken by the cell for synthesis of ribosomal and messenger RNAs, their processing, precursor formations of small and large ribosomal subunits, their transport to the cytoplasm (in eukaryotes), maturation of ribosomal subunits, assembly of active ribosomes and mRNA translation of corresponding proteins.

The equation describing the considered gene network of ribosome autocatalytic synthesis (Fig. 2) is given below:


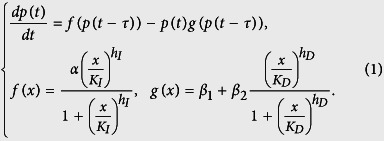


Where, *p*(*t*) – intracellular concentration of ribosomes at the current time, *f*(*x*) – a control function of ribosome synthesis rate, *g*(*x*) – a dynamic parameter of the autodegradation rate, *α* - the rate constant for synthesis, *β*_1_ – the rate constant for the constitutive degradation, *β*_2_ – the rate constant for the dynamic autodegradation, *K*_*I*_ – the efficiency constant for the autoactivation process, *K*_*D*_ – the efficiency constant for the autodegradation process, *h* – the Hill coefficient that determines the nonlinearity degree of the dynamic autodegradation.

Justification of the functions *f*(*x*) and *g*(*x*) is given in Supplementary section SI1.

In the following section we demonstrate that for certain values of the retarded argument *τ* in the equation (1) the chaotic dynamics is implemented with the following fixed set of dimensionless parameters:


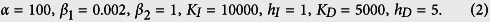


And for a given set of parameters we carry out a detailed analysis of the dynamic properties of the model (1). When selecting the set of parameter values, we proceeded from the fact that *h*_*I*_ = 1 corresponds to the simple model of synthesis initiation and *h*_*D*_ = 5 approximately corresponds to the stoichiometry of the prokaryotic proteases^29^. The values of other parameters when converted to the dimensional units are in the micromolar range of protein concentration and are charachteristic of cells (see Supplementary section SI6).

In the Supplementary section SI2 we provide an extensive analysis of the dependence of the chaotic potential of a simple model of ribosome biogenesis (1) on the parameters *h*_*I*_ and *h*_*D*_, describing nonlinear processes of ribosome synthesis and degradation.

In the Supplementary section SI4 we provide a more complex version of the model of ribosome biogenesis, taking into account the multimeric architecture of ribosomes and the protein degradation system, as well as the two stage mechanism of translation initiation; and conduct, due to a high combinatorics, an analysis of the chaotic potential of the model (equations SI1, SI11, SI12) for the 13 selective sets of parameter values (Supplementary section SI5) depending on the delay parameter *τ*.

## Results

### Stationary solutions of the model (1), (2)

In a model of ribosome biogenesis (1), the form of functions *f*(*x*) and *xg*(*x*), which control the rate of ribosome synthesis and autodegradation, suggests a zero steady state in the model (1) when *x* = 0. It is easy to determine, that the steady state is unstable when *β*_1_ > 0.

From the diagrams of the functions *f*(*x*) and *xg*(*x*) presented in Fig. 2, it follows that for the parameter values (2) the model represented by the equation (1) has a non-zero steady state solution. We find that for given values of the parameters (2) *f*(*x*) = *xg*(*x*) and is located at a 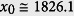 point.

According to Èl’sgol’ts & Norkin^29^, we determine the dependence of the stability by the first approximation of the found non-zero stationary solution of equation (1) on the parameter *τ, τ* ≥ 0.


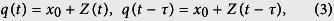


where *Z*(*t*) and Z(*t* − *τ*) are small deviations from the stationary solution.

After linearization of the right hand side of the equation (1) we obtain the linear equation with retarded argument in relation to *Z*(*t*):


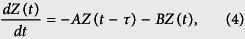


where


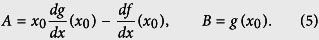


When *A* and *B* are calculated using parameters and values from the equation (2) and *x*_0_ = 1826.1, 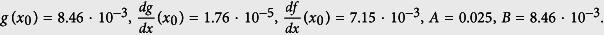 I.e., *A* > *B* > 0.

According to Èl’sgol’ts & Norkin^30^, implementation of this inequality means that when *τ*_0_ > 0 there are such values of retardation parameter *τ*, at which a stationary solution of equation (1) is either asymptotically stable (*τ* < *τ*_0_), or unstable (*τ* > *τ*_0_). The value *τ*_0_ can be found from the formula:


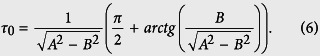


It was calculated that *τ*_0_~81.774 for the parameter values (2). That is, when *τ* ≥ 81.774, the equation (1), (2) has no stable steady states. Numerical analysis has shown that in this case solutions of the equation (1), (2) have an oscillatory dynamics that for certain values of the parameter *τ* may be periodic, quasi-periodic, or is unstable with respect to the initial data.

### Periodic solutions of the model represented by equation (1), (2)

Figure 3a demonstrates the presence of a periodic solution of equation (1), (2) when 470 ≤ *τ* ≤ 471.06. However, even at *τ* ≈ 471.07 bifurcation of a quasiperiodic solution occures, which have been shown to exist in the interval 471.07 ≤ *τ* ≤ 475.14 (Fig. 3b).

A further increase of *τ* leads to the conversion of quasi-periodic solutions into periodic solutions at first of multiplicity 5 (Fig. 3d), then 10 (Fig. 3e), 20 and 40 (Fig. 3f) at *τ* = 475.15, 487, 487.75 and 488.05, respectively.

The Poincaré map constructed from the intersection points of solution and the Poincaré plane *p*(*t*) = 2500, *p*(*t* − *τ*) > 1950 (Fig. 3c,g,h,i) demonstrate all these transitions.

### The solution of equation (1), (2) is unstable with respect to the initial data

With further value increase of the parameter *τ*, when *τ* = 488.20, the solution of equation (1), (2) becomes unstable with respect to the initial data (Fig. 4a) and the Poincaré map acquires complex fractal structure (Fig. 4c,e).

The bifurcation diagram (Fig. 5a) constructed from the intersection points of equation (1), (2) solutions and the Poincaré plane *p*(*t*) = 2500, *p*(*t* − *τ*) ≥ 1800 well demonstrates the transition to chaos when parameter *τ* value increases from 470 to 490; as well as it shows that the transition is carried out through a period doubling cascade of Feigenbaum scenario^31^,^32^.

We should note the unusual nature of the bifurcation diagram in the value ranges *τ* > 496.25 and *τ* > 510 in which we observe an abrupt increase in the area of chaotic dynamics, as reflected in Fig. 5a, with rapid borders expansion of the bifurcation diagram on the ordinate axis (arrows 1 and 2).

Quantification of the solution instability with respect to the initial data revealed the presence of only one positive Lyapunov exponent in the solutions of the equation (1), (2) (Fig. 5b) when *τ* ≤ 500, indicating that a chaos found in the investigated area of the equation (1), (2) solutions is not a hyperchaos. However, when *τ* = 900, a hyperchaos is still detected in the equation (1), (2) (Fig. 5c, d). This is evidenced by the presence of two positive senior Lyapunov exponents in the area of the equation (1), (2) solutions (Fig. 5e). Computer experiment demonstrates that the transition to hyperchaos occures from chaos.

## Biological Interpretation of the Modeling Results

The results presented above and in the Supplementary sections SI2 and SI5, were obtained for the equations in which the time, constants and variables were measured in dimensionless units. However, in order to evaluate the biological significance of the parameters for which the chaotic dynamics is observed in the studied models of ribosome biogenesis, it is necessary to assess what these parameter values may correspond to, if we turn to the natural units of measurement. When switching to the dimensional units, only values of Hill coefficients *h*_*I*_ and *h*_*D*_ do not change in the model, which are dimensionless within the meaning. To convert the dimensionless parameters of the model to dimensional parameters we used the translation algorithm, which is described in the Supplementary section SI3.

Using this algorithm, we calculated the values of parameters *K*_*I*_ and *K*_*D*_ measured in mkM, parameter *α* measured in mkM/h, parameters *β*_1_, *β*_2_ measured in h^−1^ and parameter *τ* measured in hours, for all sets of parameter values for which the models displayed chaotic dynamics (see Supplementary sections SI6).

When estimating the parameters, we proceeded from known experimental data on the half-life of ribosomal proteins, which varies from 2 to 150 hours or more in higher and lower organizms^33^,^34^; and total concentration of ribosomes in the eukaryotic cell, which we roughly estimated to be 16 μM based on the average parameters of the eukaryotic cell volume (~500 μm^3^), the number of ribosomes in the cell (~5 × 10^6^) and the ribosome molecular weight (4.2 MDa) [ http://bionumbers.hms.harvard.edu/].

The fact that the free fraction of ribosomes, which can be 15%^35^, is exposed to degradation in the cell was also considered.

The parameter values for which the models of ribosome biogenesis displayed chaotic dynamics (Fig. 5а, Fig. SI2.1 and SI5.1) are summarized in the Table SI6.1 (Supplementary section SI6).

It follows from the Table SI6.1 that the chaotic dynamics was detected for all the considered values of coefficients *h*_*I*_ and *h*_*D*_, when *h*_*D*_ ≥ 4 for the minimal model and *h*_*D*_ ≥ 2 for the expanded model. We could not find presence of chaos for *h*_*D*_ = 1 for all tested values *h*_*I*_, for both minimal and expanded models (see. Supplementary section SI2 and SI5). Given the relatively high structural complexity of the degradation system, in both prokaryotes and eukaryotes^29^,^36^, this limitation, in our view, is not critical.

With regard to the values of parameters *K*_*I*_, *K*_*D*_, which are the efficiency constants for the activation processes of ribosome synthesis and degradation, in all calculations they vary from 0.2 to 100 μМ for *K*_*I*_ and from 2 to 70 μМ for *K*_*D*_, that is, they are in the micromolar range of protein concentration characteristic of any cell.

The rate constant for synthesis, *α*, was estimated to be ~4–32 μM/h. The rate constant for the synthesis of ribosomes in the yeast cell was experimentally^37^ estimated to be ~7 μM/h, that is, of the same order as the one calculated from the model.

The delay parameter *τ*, as follows from the Table SI6.1, varies from 1.2 to 7 hours. This assessment was carried out for the 2 hours half-life of ribosomal proteins, that is, for the lower limit of the parameter value. And this means that 1.2 hour is the minimum delay at which the chaotic dynamics is realized. If the 10 hours half-life of ribosomal protein is used for the calculation of dimensional parameters, which is acceptable for both prokaryotes and eukaryotes^33^,^34^, the minimum delay value *τ* increases to 6 hours.

It is evident that the resulting estimates quite fit within the physiological borders of the parameters characteristic of molecular-genetic systems, except for the *τ* parameter, at least for prokaryotes.

## Discussion

**Figure 1:**
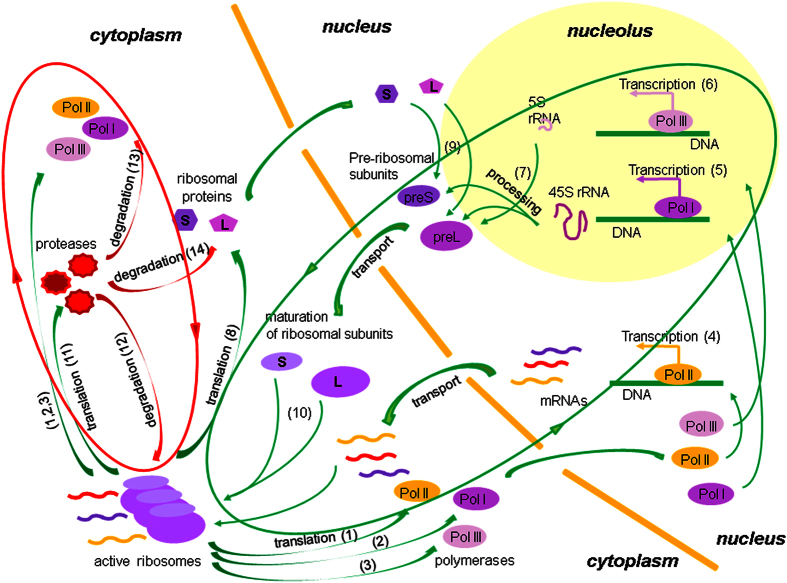
The scheme of a simple gene network of autocatalytic synthesis of ribosomes and degrading enzymes in eukaryotes. (1), (2), (3) – polymerase synthesis (Pol I, Pol II, Pol III), synthesis of enzymes ensuring the transcription of ribosomal RNA (rRNA) and messenger RNA (mRNA); (1), (4), (8) – biosynthesis of ribosomal proteins - parts of a large (L) and a small (S) ribosomal subunits; (2), (3), (5), (6) – rRNA biosynthesis; (7), (9), (10) – ribosomal RNA processing, formation of large and small ribosomal subunits, their assembly and maturation of active ribosomes; (1), (4), (11) – biosynthesis of degrading enzymes; (12), (13), (14) – degradation of proteins and ribosomes. Synthesis processes are shown with green arrows, processes of degradation – with red arrows. Processes (1–10) imitate a positive regulation loop for ribosome synthesis (green oval); processes (1, 4, 12–14) – a negative regulation loop (red oval).

**Figure 2:**
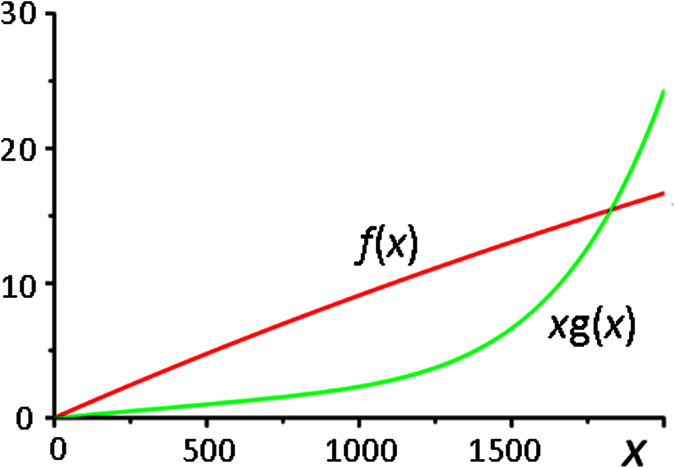
Steady state solutions in the model of ribosome biogenesis. Diagrams of the functions *f*(*x*) and *xg*(*x*) for the parameter values (2). One can see the presence of a non-zero steady state at *х*~1826.

**Figure 3:**
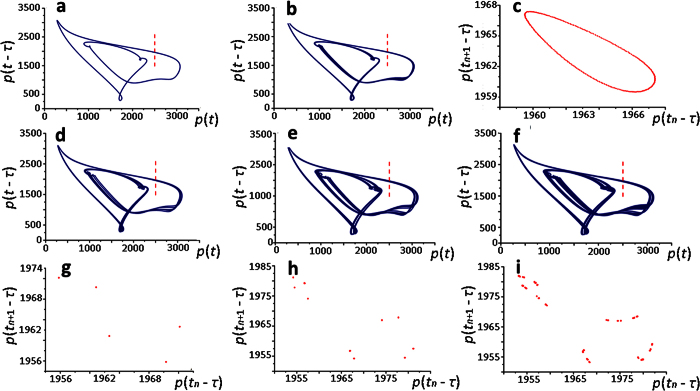
Periodic dynamics in the model of ribosome biogenesis: the dependence of the equation (1), (2) solution on the value of parameter *τ*. (**а**) periodic solution at *τ* = 471.06; (**b**) quasiperiodic solution at *τ* = 472.07; (**d**,**e**,**f**) periodic solution of multiplicity 5, 10 and 40 at *τ* = 475.15, 487, and 488.05, respectively. The Poincaré map (**с**,**g**,**h**,**i**) constructed from the intersection points of solution and the Poincaré plane *p*(*t*) = 2500, *p*(*t* − *τ*) > 1950; at *τ* = 472.14, 487 and 488.05, respectively. The Poincaré plane is marked with a vertical line.

**Figure 4:**
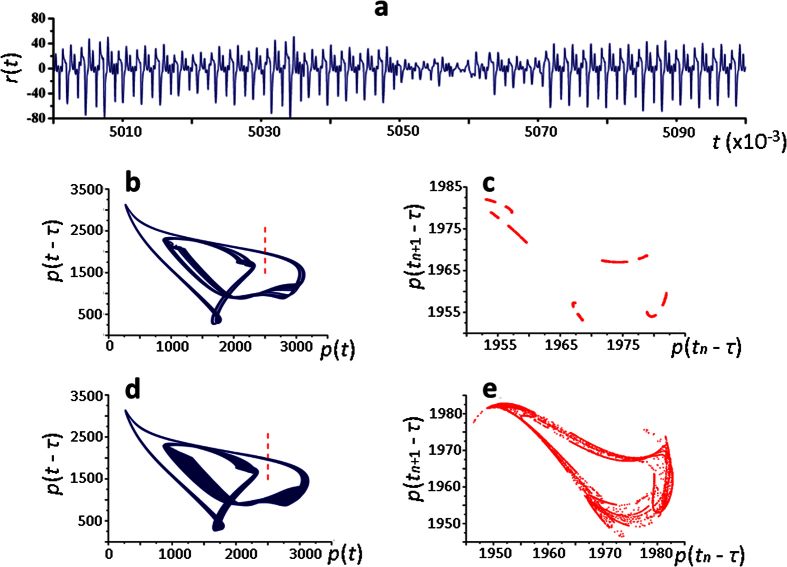
Chaotic dynamics in the model of ribosome biogenesis. (**a**) – instability of the equation (1), (2) solution with respect to the initial data: *r*(*t*) – difference between the two solutions and initial data that differ by 0.000000001 on the interval [−500.0]; (**b**,**d**) – strange attractor when *τ* = 488.20 and *τ* = 490 (the Poincaré plane is marked with a vertical line); (**c**,**e**) – corresponding Poincaré map constructed from the intersection points of the solution and the Poincaré plane *p*(*t*) = 2500, *p*(*t* − *τ*) > 1950.

In this paper, the chaotic potential of a simple genetic system of ribosome biogenesis regulation during the cell cycle was analyzed using a mathematical model. The model describes positive processes of the ribosome synthesis by themselves and the synthesis of ribosome-degrading proteins (green and red ovals in Fig. 1) with two monotonically increasing function. The analysis of functional dynamics of the equation (1), (2) has revealed the possibility of both stationary and oscillatory dynamics formations in the system of ribosome biogenesis, that for certain values of the parameters and retarded arguments was sensitive to the minor changes in the initial data, that is, it was chaotic. We stress that this system has no positive-negative feedback regulatory loops.

Nevertheless, we explain the possibility of the chaotic dynamics generation by the fact that in the system of ribosome biogenesis this circuit is present in a latent form, as the ribosome degradation process simulates negative regulation and the autocatalytic synthesis – positive regulation. An analogy can be found between this observation and the genetic systems controlled by two regulatory loops, positive and negative, for which the chaotic dynamics is well studied^16^,^17^,^18^,^19^. However, the system of ribosome biogenesis is not identical to the gene networks with positive-negative regulation of gene expression efficiency and cannot be described with the respective models^16^,^17^,^18^,^19^.

It is important to note that when the values of the parameters in the model of ribosome biogenesis are transferred to the intrinsic dimensions, they lie in the range of values typical for the living systems. In this case, as follows from the Table SI6.1, the minimum estimate of the delay parameter τ, at which the chaotic dynamics is observed, varies from 1.2 to 7 hours. Accordingly, from the model it follows that prokaryotes most likely do not experience the chaotic potential of the system, since the ~1.2–6 hours retardation of the signal is too long for prokaryotic systems.

For eukaryotes, the conclusion is not so unambiguous. In this regard, we consider the somite morphogenetic differentiation in vertebrates, the amount of stages in which depends on the type of organism and can reach 300 or more, for example, in snakes^38^,^39^. The rhythmically repeated process of somite formation in vertebrates is controlled by molecular oscillator, and the duration of each somite pair formation depends on the organism type, on the somite position relative to the longitudinal axis, and in some organisms it depends on the external conditions of their development and can range from several minutes to several hours even in organisms with a reasonably fast ontogenesis^39^. Therefore, the retardation of ~1.5–7 hours can be quite realizable for eukaryotic organisms.

It should be noted that the considered models of ribosome synthesis and degradation do not impose any strict structural restrictions on the biological system. Indeed, the value *h*_*I*_ = 1 indicates that the minimum single step model of ribosome synthesis initiation is sufficient for the formation of chaotic dynamics. The value *h*_*D*_ = 4 is suitable for the whole range of values*h*_*I*_. This complexity is not critical for the protein degradation system. It is known that in the *E.coli* cell, the main proteolytic enzyme, Lon protease, is a hexamer and the eukaryotic proteasome includes tens of proteins^29^,^36^.

And if we consider that the increase of the complexity of the model of ribosome biogenesis (see Supplementary section SI5) leads to a decrease of the *h*_*D*_ value until it reaches 2, at which the model (SI1, SI11, SI12) demonstrates chaotic dynamics, then one can assume that Hill coefficients of both simple and expanded models of ribosome biogenesis are consistent with prokaryotes and eukaryotes.

In this article we demonstrate the transition to chaos via a cascade of period-doubling bifurcations, that is, via the Feigenbaum scenario^31^,^32^. This scenario is one of the most commonly observed scenarios of transition to chaos and is well described in such biological systems as the environmental population models^40^,^41^,^42^,^43^,^44^; the biophysical models of the electrical activity dynamics of neurons and pancreatic β-cells^45^,^46^,^47^,^48^; the models of intracellular oscillations of calcium concentrations^49^ and NADH^12^ concentrations; as well as in the molecular genetic systems – the model of alternative splicing^18^. Hence, in fact, this scenario is observed at all levels of life organization – from molecular to population level.

**Figure 5:**
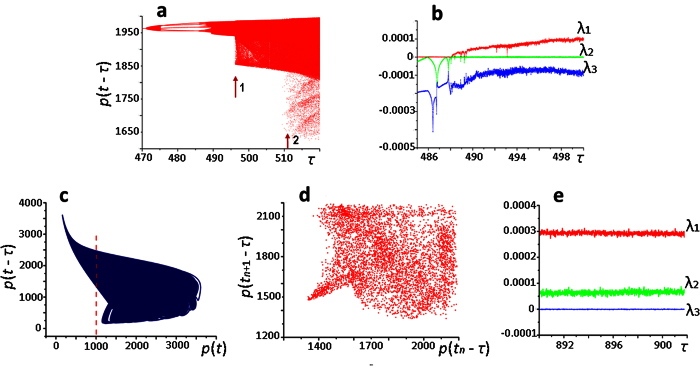
Transition to chaos and hyperchaos in the equation (1), (2). (**а**) – bifurcation diagram constructed from the intersection points of equation (1), (2) solutions and the Poincaré plane *p*(*t*) = 2500, *p*(*t* − *τ*) ≥ 1800 (an abrupt increase in the field of chaotic dynamics is indicated by arrows 1 and 2); (**b**) – values of the senior Lyapunov exponents for solutions of equation (1), (2) when *τ* > 488.20; (**c**) – hyperchaotic attractor at *τ* = 900 in the plane (*p*(*t*), *p*(*t* − *τ*)); (**d**) – unilateral Poincaré map, constructed from the intersection points of the solution and the Poincaré plane *p*(*t*) = 1000 (the Poincaré plane is marked with a vertical line); (**e**) – assessment of three senior Lyapunov exponents in the hyperchaos area.

It should be noted that in the system of ribosome autocatalytic synthesis both chaos and hyperchaos are observed. The peculiarity of the hyperchaotic attractor, in contrast to the chaotic attractor, is the existence of more than one positive Lyapunov exponent (Fig. 5e). This is a more complex dynamic behavior of the system, which has been recently confirmed for the activity of the mollusc *Clione limacina* sensory neurons. According to the authors^50^, the hyperchaotic dynamics may be a mechanism that reflects its food-search strategy. But the reason why hyperchaos is more suitable for this purpose remains unclear.

In the presented model of ribosome biogenesis the hyperchaotic dynamics arises from the chaotic dynamics when retarded argument increases. From a biological point of view, the feasibility of this phenomenon at the intracellular level is not obvious, as in the model of molecular-genetic system of ribosome biogenesis hyperchaos is observed outside the physiological values of the retarded argument. However, the very fact of its discovery suggests such a possibility in the molecular-genetic systems for which such values of retarded argument may well be physiological.

From a mathematical point of view, the received result is original, since it demonstrates the presence of a chaotic potential in the system described with two monotonically increasing functions with one delayed argument. Earlier, similar results were shown for the systems described with unimodal functions with delayed argument and for the systems described with monotonically decreasing functions with two delayed arguments^15^,^17^,^18^,^19^,^28^; in the first case a chaotic potential was found for large Hill coefficients (≥10)^28^, and in the second – for the case of significant difference between the values of delayed arguments^17^,^18^,^19^,^28^, that seems an unlikely case for molecular-genetic systems.

In conclusion, it should be noted that the present work is the first to theoretically demonstrate the possibility of chaotic dynamics generation in the system of ribosome biogenesis in the biologically relevant areas of functioning of the eukaryotic cell, which depends only on the internal features of the translation system functioning, in the absence of any regulatory impacts. And that means that in the evolutionary aspect the cell faced the problem of metabolic instability rather early, even at the origin of the translation system. Nevertheless, at the molecular-genetic level of the biological organization, as opposed to the organism and population levels^51^,^52^,^53^,^54^,^55^,^56^, until now there have been no experimentally proven examples of chaotic dynamics *in vivo*.

The lack of experimental evidence of the chaos generation at the intracellular level *in vivo* may indicate that during evolution the cell has found a solution to this problem - the possibility of stabilizing the system in the presence of chaos-generating factors. However, we do not know yet how the cell has solved the problem.

## Methods

### Calculation method

Integration of the equation was performed by the steps method^30^ using the original program written in Fortran. Calculations were performed on a computing complex of the Information and Computing Center, Novosibirsk State University (http://www.nusc.ru).

### Determination of the type of the oscillatory dynamics

The type of the oscillatory dynamics can be visualized by the characteristic Poincaré map: circular trajectory will generate the Poincare map that consists of a finite number of points over which the trajectory will go in a certain order; the quasicyclic trajectory is displayed on the plane as a combination of closed curves; the Poincaré map of a strange attractor is an infinite set of unordered points. Description of the Poincaré map construction is given below.

The criterion of the chaotic state of the oscillatory dynamics was considered to be the sensitivity to initial data of the equation (1) solution, which is detected by calculating the difference between the two trajectories of one variable, calculated with two identical models that start at zero time point with the initial functions that vary by a “small” value. If, as we iterate over values, the difference becomes comparable with the fluctuation amplitude, the dynamics is concluded to be sensitive to initial data.

To quantify the instability of the solution with respect to the initial data, the Lyapunov exponents were used, which allow to explicitly identify the motion pattern in the system. The calculation method is given below.

With the correct choice of the plane position, **the Poincaré map** gives us a clear idea of the type of oscillatory motion. The map is constructed as follows: we choose a part of the plane, which intersects the trajectory of the equation (1) solution at a certain point *х*_0_. The first point of the trajectory intersection with the selected part of the plane, which follows after the point *х*_0_, we shall designate *х*_1_ = *P*(*х*_0_). Then we define the second point of trajectory intersection with the selected part of the plane, *х*_2_ = *P*(*х*_1_), and so on. The set of intersection points is named the Poincaré map.

**The Lyapunov exponents** characterize the degree of exponential growth (or decay) of disturbances near the attractor trajectory. A spectrum of Lyapunov exponents is equal in number to the dimensionality of the phase space. For systems of equations with retarded arguments, for which the phase space is infinite, the number of Lyapunov exponents is also infinite. However, only a few senior Lyapunov exponents have a significant impact. The presence in the spectrum of at least one positive Lyapunov exponent is a measure of chaos. If a chaotic dynamics has two or more positive Lyapunov exponents, such a dynamics is identified to be hyperchaotic^57^.

Let us give a description of the method of calculation of several senior Lyapunov exponents for the retarded equation based on a modification of a known method of Benettin^58^,^59^. Let us consider the retarded equation:


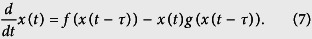


Let the initial distribution *x*(*t*) be given in the interval (−*τ*, 0), with a uniform partition with the step *h*


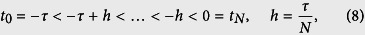


Simultaneously with the equation (7) we consider the *k* number of perturbed equations


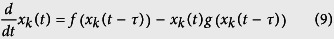


Let the vector with the components determined as values of the function *x*(*t*) at the grid points be 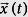:


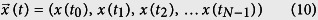


In addition, let us introduce vectors 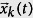 for disturbances: 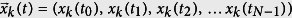. The initial distributions for the perturbation vectors are given in such a way that 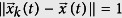. Let us integrate the equation (7) simultaneously with the equations (9) on the segment of *τ* length and subject the vectors 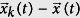 to the Gram - Schmidt orthogonalization with the transition to the orthonormal basis of vectors 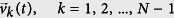. On the next segment of *τ* length we specify the initial distribution for the disturbances vectors as 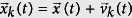.

After the integration of the equations (7), (9) the first step procedure is repeated, and so on. This procedure is performed a sufficiently large number of *M* times. At the same time the sums 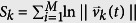 are calculated, which include the norms of the vectors after orthogonalization, but before renormalization.

An evaluation of the Lyapunov exponents is obtained as follows:


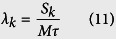


After a sufficiently large number of steps *M* the values *λ*_*k*_ will converge. In our calculations, the mean values and standard deviations when *Mτ* > 10^6^ were used to assess the senior Lyapunov exponents.

We shall note that the solutions of retarded equations (7) are invariant with respect to the reference time, which corresponds to the disturbance which does not increase and does not decay, therefore, there must be at least one zero parameter in the spectrum of Lyapunov exponents.

## Additional Information

**How to cite this article**: Likhoshvai, V. A. *et al*. Chaos and Hyperchaos in a Model Of Ribosome Autocatalytic Synthesis. *Sci. Rep.* **6**, 38870; doi: 10.1038/srep38870 (2016).

**Publisher's note:** Springer Nature remains neutral with regard to jurisdictional claims in published maps and institutional affiliations.

## Supplementary Material

Supplementary Information

## References

[b1] Hadjiolov A. A. Biogenesis of ribosomes in eukaryotes. Subcell. Biochem. 7, 1–80 (1980). 7003820 10.1007/978-1-4615-7948-9_1

[b2] Larson D. E., Zahradka P. & Sells B. H. Control points in eucaryotic ribosome biogenesis. Biochem. Cell. Biol. 69, 5–22 (1991). 2043343 10.1139/o91-002

[b3] Nomura M. Regulation of ribosome biosynthesis in *Escherichia coli* and *Saccharomyces cerevisiae*: diversity and common principles. J. Bacteriol. 181, 6857–6864 (1999). 10559149 10.1128/jb.181.22.6857-6864.1999 PMC94158

[b4] Deutscher M. P. Degradation of stable RNA in bacteria. J. Biol. Chem. 278, 45041–45044 (2003). 12941949 10.1074/jbc.R300031200

[b5] Kaczanowska M. & Rydén-Aulin M. Ribosome biogenesis and the translation process in *Escherichia coli*, Microbiol. Mol. Biol. Rev. 71, 477–494 (2007). 17804668 10.1128/MMBR.00013-07 PMC2168646

[b6] Henras A. K. . The post-transcriptional steps of eukaryotic ribosome biogenesis. Cell. Mol. Life. Sci. 65, 2334–2359 (2008). 18408888 10.1007/s00018-008-8027-0 PMC11131730

[b7] Zundel M. A., Basturea G. N. & Deutscher M. P. Initiation of ribosome degradation during starvation in *Escherichia coli*. RNA 15, 977–983 (2009). 19324965 10.1261/rna.1381309 PMC2673067

[b8] Woolford J. L. Jr. & Baserga S. J. Ribosome biogenesis in the yeast *Saccharomyces cerevisiae*. Genetics 195, 643–681 (2013). 24190922 10.1534/genetics.113.153197 PMC3813855

[b9] Chen S. S. & Williamson J. R. Characterization of the ribosome biogenesis landscape in *E. coli* using quantitative mass spectrometry. J. Mol. Biol. 425, 767–779 (2013). 23228329 10.1016/j.jmb.2012.11.040 PMC3568210

[b10] Thomson E., Ferreira-Cerca S. & Hurt E. Eukaryotic ribosome biogenesis at a glance. J. Cell. Sci. 126, 4815–4821 (2013). 24172536 10.1242/jcs.111948

[b11] Lafontaine D. L. Noncoding RNAs in eukaryotic ribosome biogenesis and function. Nat. Struct. Mol. Biol. 22, 11–19 (2015). 25565028 10.1038/nsmb.2939

[b12] Martinez de la Fuente I., Martinez L. & Veguillas J. Dynamic behavior in glycolytic oscillations with phase shifts. Biosystems 35, 1–13 (1995). 7772719 10.1016/0303-2647(94)01473-k

[b13] Martinez de la Fuente I., Martinez L., Veguillas J. & Aguirregabiria J. M. Quasiperiodicity route to chaos in a biochemical system. Biophys. J. 71, 2375–2379 (1996). 8913578 10.1016/S0006-3495(96)79431-6 PMC1233727

[b14] Goldbeter A. . From simple to complex oscillatory behavior in metabolic and genetic control networks. Chaos 11, 247–260 (2001). 12779458 10.1063/1.1345727

[b15] Bastos de Figueiredo J. C., Diambra L., Glass L. & Malta C. P. Chaos in two-looped negative feedback systems. Phys. Rev. E. Stat. Nonlin. Soft. Matter. Phys. 65, 051905 (2002). 12059591 10.1103/PhysRevE.65.051905

[b16] Zhang Z. . Chaotic motifs in gene regulatory networks. PLoS One 7, e39355 (2012). 22792171 10.1371/journal.pone.0039355 PMC3391214

[b17] Likhoshvai V. A., Fadeev S. I., Kogai V. V. & Khlebodarova T. M. On the chaos in gene networks. J. Bioinform. Comput. Biol. 11, 1340009 (2013). 23427991 10.1142/S021972001340009X

[b18] Likhoshvai V. A., Kogai V. V., Fadeev S. I. & Khlebodarova T. M. Alternative splicing can lead to chaos. J. Bioinform. Comput. Biol. 13, 1540003 (2015). 25556917 10.1142/S021972001540003X

[b19] Suzuki Y., Lu M., Ben-Jacob E. & Onuchic J. N. Periodic, quasi-periodic and chaotic dynamics in simple gene elements with time delays. Sci. Rep. 6, 21037 (2016). 26876008 10.1038/srep21037 PMC4753448

[b20] Leloup J. C. & Goldbeter A. Chaos and birhythmicity in a model for circadian oscillations of the PER and TIM proteins In Drosophila. J. Theor. Biol. 198, 445–459 (1999). 10366496 10.1006/jtbi.1999.0924

[b21] Leloup J. C., Gonze D. & Goldbeter A. Limit cycle models for circadian rhythms based on transcriptional regulation In Drosophila and Neurospora. J. Biol. Rhythms 14, 433–448 (1999). 10643740 10.1177/074873099129000948

[b22] Lloyd A. L. & Lloyd D. Hypothesis: the central oscillator of the circadian clock is a controlled chaotic attractor. Biosystems 29, 77–85 (1993). 8374069 10.1016/0303-2647(93)90085-q

[b23] Ciliberto A., Novak B. & Tyson J. J. Mathematical model of the morphogenesis checkpoint in budding yeast. J. Cell. Biol. 163, 1243–1254 (2003). 14691135 10.1083/jcb.200306139 PMC2173725

[b24] Gonze D., Halloy J., Leloup J. C. & Goldbeter A. Stochastic models for circadian rhythms: effect of molecular noise on periodic and chaotic behavior. C. R. Biol. 326, 189–203 (2003). 12754937 10.1016/s1631-0691(03)00016-7

[b25] Gérard C. & Goldbeter A. From simple to complex patterns of oscillatory behavior in a model for the mammalian cell cycle containing multiple oscillatory circuits. Chaos 20, 045109 (2010). 21198121 10.1063/1.3527998

[b26] Romond P. C., Rustici M., Gonze D. & Goldbeter A. Alternating oscillations and chaos in a model of two coupled biochemical oscillators driving successive phases of the cell cycle. Ann. NY Acad. Sci. 879, 180–193 (1999). 10415827 10.1111/j.1749-6632.1999.tb10419.x

[b27] Sprott J. C. Chaotic dynamics on large networks. Chaos 18, 023135 (2008). 18601501 10.1063/1.2945229

[b28] Mackey M. C. & Glass L. Oscillation and chaos in physiological control systems. Science 197, 287–289 (1977). 267326 10.1126/science.267326

[b29] Park S. C. . Oligomeric structure of the ATP-dependent protease La (Lon) of *Escherichia coli*. Mol. Cells 21, 129–134 (2006). 16511355

[b30] El’sgol’ts L. E. & Norkin S. B. Introduction to the theory of differential equations with deviating argument. (Nauka, 1971).

[b31] Feigenbaum M. J. The Universal Metric Properties of Nonlinear Transformations. J. Stat. Phys. 21, 669–706 (1979).

[b32] Feigenbaum M. J. Universal Behavior in Nonlinear Systems. Los Alamos Science 1, 4–27 (1980).

[b33] Doherty M. K., Hammond D. E., Clague M. J., Gaskell S. J. & Beynon R. J. Turnover of the human proteome: determination of protein intracellular stability by dynamic SILAC. J. Proteome Res. 8, 104–112 (2009). 18954100 10.1021/pr800641v

[b34] Jayapal K. P. . Multitagging proteomic strategy to estimate protein turnover rates in dynamic systems. J. Proteome Res. 9, 2087–2097 (2010). 20184388 10.1021/pr9007738

[b35] Arava Y. . Genome-wide analysis of mRNA translation profiles in *Saccharomyces cerevisiae*. Proc. Natl. Acad. Sci. USA. 100, 3889–3894 (2003). 12660367 10.1073/pnas.0635171100 PMC153018

[b36] Kim H. M., Yu Y. & Cheng Y. Structure characterization of the 26S proteasome. Biochim. Biophys. Acta 1809, 67–79 (2011). 20800708 10.1016/j.bbagrm.2010.08.008 PMC3010250

[b37] Huber A. . Sch9 regulates ribosome biogenesis via Stb3, Dot6 and Tod6 and the histone deacetylase complex RPD3L. EMBO J. 30, 3052–3064 (2011). 21730963 10.1038/emboj.2011.221 PMC3160192

[b38] Richardson M. K., Allen S. P., Wright G. M., Raynaud A. & Hanken J. Somite number and vertebrate evolution. Development 125, 151–160 (1998). 9486789 10.1242/dev.125.2.151

[b39] Gomez C. . Control of segment number in vertebrate embryos. Nature 454, 335–339 (2008). 18563087 10.1038/nature07020

[b40] Ruxton G. D. & Rohani P. Population floors and the persistence of chaos in ecological models. Theor. Popul. Biol. 53, 175–183 (1998). 9682024 10.1006/tpbi.1998.1312

[b41] Frisman E. Y., Revutskaya O. L. & Neverova G. P. Complex dynamic modes of a population with age and sex structures. Dokl. Biol. Sci. 431, 152–156 (2010). 20506858 10.1134/s0012496610020225

[b42] Frisman E. Y., Neverova G. P. & Revutskaya O. L. Complex dynamics of the population with a simple age structure. Ecological Modelling 222, 1943–1950 (2011).

[b43] Frisman E. Y., Neverova G. P., Kulakov M. P. & Zhigalskii O. A. Changing the Dynamic Modes in Populations with Short Life Cycle: Mathematical Modeling and Simulation. Math. Biol.Bioinf. 9, 414–429 (2014).

[b44] Neverova G. P. & Frisman E. Ya. Dynamic regimes of local homogeneous population with delayed density dependence, Math. Biol. Bioinf. 10, 309–324 (2015).

[b45] Aihara K., Matsumoto G. & Ikegaya Y. Periodic and non-periodic responses of a periodically forced Hodgkin-Huxley oscillator. J. Theor. Biol. 109, 249–269 (1984). 6482467 10.1016/s0022-5193(84)80005-3

[b46] Komendantov A. O. & Kononenko N. I. Deterministic chaos in mathematical model of pacemaker activity in bursting neurons of snail. Helix pomatia. J. Theor. Biol. 183, 219–230 (1996). 8977879 10.1006/jtbi.1996.0215

[b47] Mosekilde E., Lading B., Yanchuk S. & Maistrenko Y. Bifurcation structure of a model of bursting pancreatic cells. Biosystems 63, 3–13 (2001). 11595325 10.1016/s0303-2647(01)00142-3

[b48] Jia B., Gu H., Li L. & Zhao X. Dynamics of period-doubling bifurcation to chaos in the spontaneous neural firing patterns. Cogn. Neurodyn. 6, 89–106 (2012). 23372622 10.1007/s11571-011-9184-7 PMC3253168

[b49] Shen P. & Larter R. Chaos in intracellular Ca2+ oscillations in a new model for non-excitable cells. Cell Calcium 17, 225–232 (1995). 7621534 10.1016/0143-4160(95)90037-3

[b50] Varona P., Rabinovich M. I., Selverston A. I. & Arshavsky Y. I. Winnerless competition between sensory neurons generates chaos: A possible mechanism for molluscan hunting behavior. Chaos 12, 672–677 (2002). 12779595 10.1063/1.1498155

[b51] Qu Z. Chaos in the genesis and maintenance of cardiac arrhythmias. Prog. Biophys. Mol. Biol. 105, 247–257 (2011). 21078337 10.1016/j.pbiomolbio.2010.11.001 PMC3047604

[b52] Korn H. & Faure P. Is there chaos in the brain? II. Experimental evidence and related models. C. R. Biol. 326, 787–840 (2003). 14694754 10.1016/j.crvi.2003.09.011

[b53] Constantino R. F., Desharnais R. A., Cushing J. M. & Dennis B. Chaotic dynamics in an insect population. Science 275, 389–391 (1997). 8994036 10.1126/science.275.5298.389

[b54] Becks L., Hilker F. M., Malchow H., Jürgens K. & Arndt H. Experimental demonstration of chaos in a microbial food web. Nature 435, 1226–1229 (2005). 15988524 10.1038/nature03627

[b55] Yip K. P. & Holstein-Rathlou N. H. Chaos and non-linear phenomena in renal vascular control. Cardiovasc. Res. 31, 359–370 (1996). 8681323

[b56] Gu H. Experimental observation of transition from chaotic bursting to chaotic spiking in a neural pacemaker. Chaos 23, 023126 (2013). 23822491 10.1063/1.4810932

[b57] Rossler O. E. An equation for hyperchaos. Phys. Lett. A 71, 155–157 (1979).

[b58] Benettin G., Galgani L. & Strelcyn J.-M. Kolmogorov entropy and numerical experiments. Phys. Rev. A 14, 2338–2345 (1976).

[b59] Benettin G., Galgani L., Giorgilli A. & Strelcyn J.-M. Lyapunov exponents for smooth dynamical systems and Hamiltonian systems; a method for computing all of them, Part I: Theory. Part 2: Numerical applications. Meccanica 15, 9–30 (1980).

