# Supplementary material for: Chaos and Hyperchaos in a Model of Ribosome Autocatalytic Synthesis
Source: Sci Rep. 2016 Dec 12;6:38870. doi: 10.1038/srep38870 (PMC5151018; doi:10.1038/srep38870)
Supplement: Supplementary Information [file srep38870-s1.pdf]

CHAOS AND HYPERCHAOS IN A MODEL OF RIBOSOME AUTOCATALYTIC SYNTHESIS

Vitaly A. Likhoshvai, Vladislav V. Kogai, Stanislav I. Fadeev, Tamara M. Khlebodarova

Supplementary Information

SI 1. Simple model of autocatalytic synthesis and degradation of ribosomes.

In this section we present a justification of the forms of functions *f(x)* and *g(x)* in the equations of the model (1) (2) of the main text of the article.

Let us denote the ribosome concentration by *p*. The ribosome synthesis rate we denote by *f*, while the parameter of the ribosome degradation rate - by g. Then, based on the biochemical nature of the processes which occur within a cell, a simple model for describing the time rate of the ribosome concentration change can be described with the equation

$$\frac{dp}{dt} = f - g \times p\,. \qquad \text{(SI1)}$$

Functions *f* and *g* describe the processes of ribosome synthesis and degradation, which are schematically depicted in Fig. 1 of the main text.

Let us set the form of *f*. The process of ribosome generation in a narrow sense starts from the initiation stages of rRNA and protein synthesis (green oval in Fig. 1 in the main text of the article). In a broad sense, another starting point should be added - the initiation of synthesis of RNAs encoding protein structures.

Then, new copies of ribosomes occur in the cytoplasm through a chain (some steps can occur in parallel) of successive stages:elongation, termination of transcription, splicing, mRNA transport from the nucleus to the cytoplasm, initiation, elongation, termination of translation of ribosomal proteins and RNA polymerases 1,2 and 3, transport of RNA polymerases 1,3 and ribosomal proteins from the cytoplasm to the nucleolus, ribosome assembly, transport of RNA polymerase 2 to the nucleus, transport of ribosomes to the cytoplasm.

Since all proteins including RNA polymerases are synthesized by ribosomes, we come to the conclusion that for the simple model (A1) *f* is a function of the variable *p*.

As it takes some time from the initiation of RNA and protein synthesis until the onset of the active ribosome, we conclude that ribosomal components, which appeared at the current time point *t*, have been initiated at some previous time point t-τ_1. That is, function *f* is a function of the variable $p(t-\tau_1)$: $f = f(p(t-\tau_1))$.

Based on the fact that the ribosome represents, in essence, an autocatalytic machinery we find the type of *f* dependence on *p*. Therefore, if we ignore the negative regulation mechanisms, the higher the number of ribosomes in a cell, the higher the rate of their synthesis. Hence, *f* represents a monotonically increasing nonnegative function of the variable *p*. And, due to the biochemical laws, the *f* function is bounded from above.

To reproduce such qualitative properties the Hill function is used

$$f(p) = \alpha \frac{\left(\frac{p}{K_I}\right)^{h_I}}{1 + \left(\frac{p}{K_I}\right)^{h_I}} \qquad \text{(SI2)}$$

Where, α - generalized rate constant for synthesis, K_I – generalized efficiency constant for autoactivation, h_I – Hill coefficient determining the nonlinearity degree of the impact of the number of ribosomes on the synthesis rate.

When h_I=1 the f function acquires the simplest form, which is identical to the classic function of Michaelis-Menten (Michaelis, Menten, 1913; 2013) and was used for the calculation of the model (2) in the main text.

The graph of the f function for various values of the parameters K_I and h_I is shown in Fig. SI1.1.

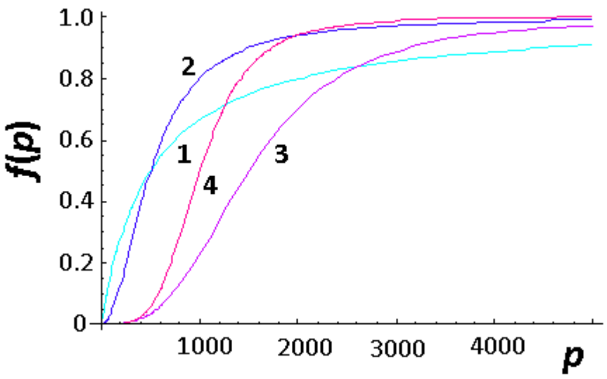

Figure SI1.1: The graph of the f function depending on the values of the parameters K_I and h_I. Curve 1 - K_I=500, h_I=1, curve 2 - K_I=500, h_I=2, curve 2 - K_I=1500, h_I=3, curve 4 - K_I=1000, h_I=4.

Based on the analysis of the synthesis of the degradation system components (red oval in Fig.1 in the main text of the article), which is generally identical to that described above, we conclude that it can be roughly described with the function:

$$g = \beta_1 + \beta_2 \frac{\left(\frac{p(t-\tau_2)}{K_D}\right)^{h_D}}{1 + \left(\frac{p(t-\tau_2)}{K_D}\right)^{h_D}}. \qquad \text{(SI3)}$$

It consists of two members. The first member (β_1) is a constant that describes the constitutive rate of the functionality loss by the ribosomes. The second member describes the dynamic process of degradation, which is accomplished by the degradation system. Up to notation, this member is identical to the f function. This member consists of three parameters: β_2 – rate constant for the dynamic autodegradation, K_D – efficiency constant for the autodegradation, h_D – Hill coefficient determining the nonlinearity degree of the impact of the number of ribosomes on the rate of the dynamic degradation. The g function, like the f function, is a nonnegative monotonically increasing and bounded above function of the argument p, τ_2 is the time spent on the synthesis of a new unit of the degradation system.

In conclusion, we emphasize once more that functions f and g represent the most common qualitative traits of the ribosome synthesis-degradation machinery.

These include: a generic starting point of the synthesis of ribosomal components; the appearance of a newly synthesized active ribosome some time after the beginning of the synthesis; monotonic increase in the activity of synthesis and degradation with increasing concentration of ribosomes.

The description of these features was carried out using the Hill functions, which allow to describe complex processes in the simplest way (Likhoshvai, Ratushny, 2007). Therefore, the model (2) in the main text is among the simplest possible models which may be used to describe the biological system of autocatalytic synthesis and degradation of ribosomes.

The genetic circuit corresponding to the considered model of ribosome biogenesis is shown in Figure SI1.2.

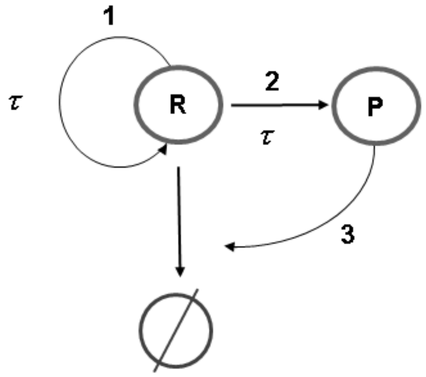

Figure SI1.2: The genetic circuit of the regulation of autocatalytic synthesis (1) and degradation (2) of ribosomes.
R – ribosomes, P – proteases, τ – time of synthesis of ribosomal proteins and degrading enzymes.

More complex variants of the model of ribosome biogenesis will be discussed in the Supplementary section SI 4.

References

1. Michaelis, L. & Menten, M.M. The kinetics of invertin action. 1913. *FEBS Lett.* **587**, 2712-2720 (2013).
2. Likhoshvai, V. & Ratushny, A. Generalized Hill function method for modeling molecular processes. *J. Bioinform. Comput. Biol.* **5**, 521-531 (2007)

SI 2. Dependence of the dynamic properties of the model, equations (SI1) - (SI3), on the parameters h_I, and h_D.

The chaotic potential of the model (equations SI1-SI3) depending on the values of parameters h_I and h_D, describing nonlinearity of the ribosome synthesis and degradation, is analyzed is this section.

Due to the fact that for each set of parameter values independent calculation is required, it is not possible to run over all variants. Therefore, we present the analysis of the dynamic properties of the model (equations SI1-SI3) for the selective parameter values.

For simplicity, we used equal values of parameters $\tau_1=\tau_2=\tau$ and integer values of the Hill coefficients (h_I, h_D). We could not find chaotic dynamics in the model (equations SI1-SI3) for the following combinations of values of the coefficients h_I and h_D: (1, 1), (1, 2), (1,3), (2,1), (2,2), (3,1), (3,2), (4,1), (4,2), (5,1), (10,1).

That is, the analysis of the modeling results have shown that for h_I=1 the chaotic dynamics was detected when $h_D \geq 4$, for h_I=2,3,4 – when $h_D \geq 3$, and for h_I=5,10 the chaotic dynamics was already observed when $h_D \geq 2$. It can be noted that with an increase in the value h_I=1 the border of "chaos" at h_D was reduced to a value of 2. In this case, the chaotic dynamics could not be found for h_D=1.

To illustrate the foregoing, we provide the bifurcation diagrams calculated for the four different values of the parameters h_I and h_D depending on the delay parameter τ (Fig. SI2.1).

It can be noted that all diagrams have individual characteristics, but in all cases there are chaotic areas and regularity areas. In some areas a doubling of cycles can be observed.

A more careful analysis of the chaotic properties of the model (SI1-SI3) were carried out for the parameter values α=100, β_1=0.002, β_2=1, K_I=10000, h_I=1, K_D=5000, h_D=5 and the results of the analysis were presented in the main text of the article.

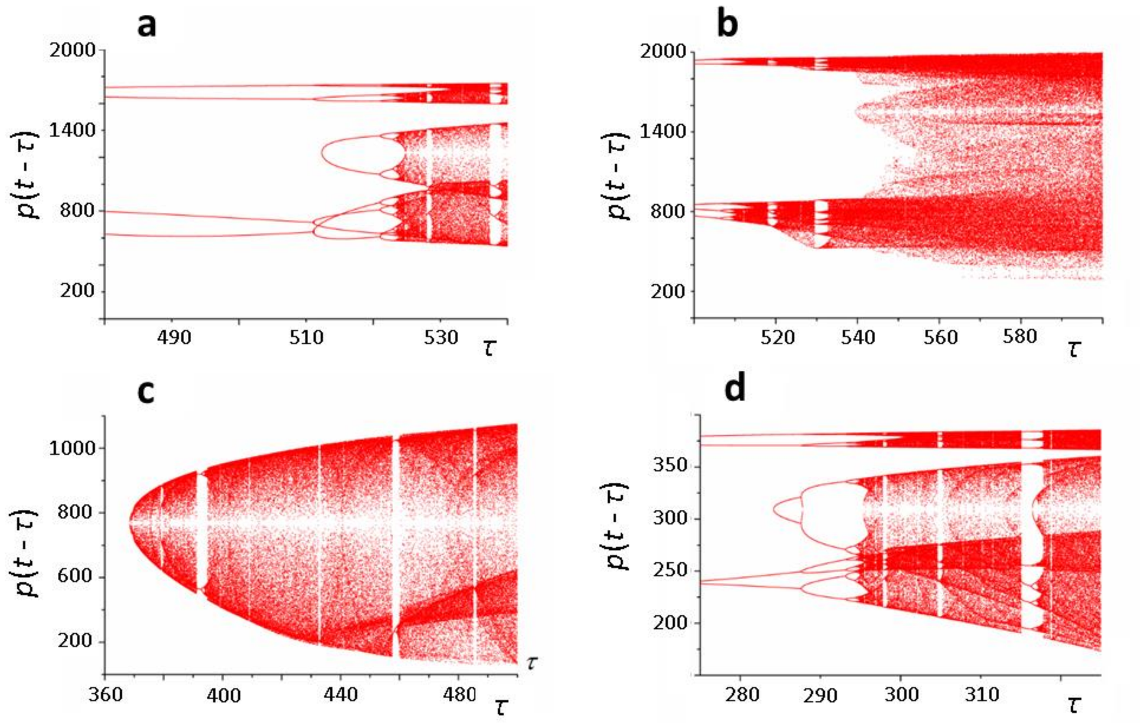

Figure SI2.1: Bifurcation diagrams constructed from the intersection points of the equation (SI1-SI3) solutions with the Poincaré plane.

(a) Poincaré plane $p(t)$ = 2500, parameter values $h_I = 1, h_D = 4$; (b) Poincaré plane $p(t)$ = 2500, parameter values $K_I = 1000, h_I = 1, h_D = 5$; (c) Poincaré plane $p(t)$ = 2500, parameter values $h_I = 2, h_D = 3$; (d) Poincaré plane $p(t)$ = 500, parameter values $K_D = 1000, h_I = 2, h_D = 4$. Values of other parameters $\alpha = 20, \beta_1 = 0.002, \beta_2 = 1, K_I = 500, K_D = 5000$.

SI 3. Algorithm for converting the dimensionless parameters of the model to the dimensional units.

To assess the compliance between the biological systems and the parameter values for which the chaos is observed, it is necessary to convert the dimensionless values of the parameters to the dimensional units. The following algorithm is used.

For a specific set of parameter values a dynamic trajectory is calculated. Since we are interested in the chaotic dynamics, calculation is carried out long enough for the trajectory to enter the chaotic attractor.

Then the mean values on the attractor of the variable p (denoted by $\bar{p}$) and the dynamic degradation parameter g (denoted by $\bar{g}$) are calculated.

$\bar{p} = P_{cell}$, $\frac{\ln 2}{\bar{g}} = T_{1/2,rib}$, where P_{rib} - average concentration of free ribosomes in a cell, $T_{1/2,rib}$ - half life of free ribosomes in a cell. We consider these cell parameters as known values.

Then the dimensionless parameters of the model can be converted to the dimensional units using the following formulas:

$$\tau_{\dim} = \frac{\bar{g}}{\ln 2}\tau T_{1/2,rib}, \beta_{\dim,1} = \frac{\ln 2}{T_{1/2,rib}\bar{g}}\beta_1, \beta_{\dim,2} = \frac{\ln 2}{T_{1/2,rib}\bar{g}}\beta_2,$$

$$K_{\dim,I} = \frac{K_I}{\bar{p}}P_{Rib}, K_{\dim,D} = \frac{K_D}{\bar{p}}P_{Rib}, \alpha_{\dim} = \frac{\alpha P_{Rib}}{\bar{p}\bar{g}T_{1/2,rib}}.$$

SI 4. Model of autocatalytic synthesis-degradation of ribosomes based on the multimeric architecture of ribosomes and the two stage mechanism of translation initiation.

In the section SI1 we described the simplest variant of a mathematical model for describing the system of synthesis-degradation of ribosomes. In it, we did not consider any structural details of the ribosome synthesis and degradation machinery.

In this section, we will consider a more complex variant of the model (SI1). In it, such forms of functions *f* and *g* were used, which take into account the multimeric architecture of ribosomes and proteasomes, as well the two stage mechanism of translation initiation.

According to this, variable *p* represents a generalized subunit of ribosome in the model (SI1). Let us assume that the protein synthesis rate is proportional to the concentration of the ribosomal initiation complex. Its value (*d*) is calculated on the basis of a system of two bimolecular reactions:

$$a + b_1 \underset{k_{12}}{\overset{k_{11}}{\rightleftharpoons}} c, c + b_2 \underset{k_{22}}{\overset{k_{21}}{\rightleftharpoons}} d \ . \qquad \text{(SI4)}$$

From (SI4) write out the chemical system of differential equations, neglecting the changes of b_1 and b_2:

$$\begin{cases} a' = -\left(k_{11}ab_1 - k_{12}c\right), \\ c' = \left(k_{11}ab_1 - k_{12}c\right) - \left(k_{21}cb_2 - k_{22}d\right), \\ d' = \left(k_{21}cb_2 - k_{22}d\right). \end{cases} \qquad \text{(SI5)}$$

Equate the right hand side of (SI5) to zero.

Taking into account the conservation law, obtain a system of equations:

$$\begin{cases} \left(k_{11}ab_1 - k_{12}c\right) = 0, \\ \left(k_{21}cb_2 - k_{22}d\right) = 0' \\ a + c + d = a_0 + c_0 + d_0. \end{cases}$$

Find a solution

$$\begin{cases} a\left(1 + \frac{k_{11}}{k_{12}}b_1 + \frac{k_{11}k_{21}}{k_{12}k_{22}}b_1 b_2\right) = a_0 + c_0 + d_0, \\ c\frac{k_{11}}{k_{12}}b_1 a, \\ d = \frac{k_{11}k_{21}}{k_{12}k_{22}}b_1 b_2 a. \end{cases} \qquad \text{(SI6)}$$

Assume that b_1 and b_2 have a meaning of a concentration of free generalized ribosomal subunits (p), $m=\left(a_0+c_0+d_0\right)$ - a meaning of RNA concentration; and denote $K_1=\frac{k_{12}}{k_{11}}, K_2=\frac{k_{22}}{k_{21}}$. Obtain the following equation for the synthesis initiation rate of the i protein

$$\left\{ d_i\left(p(t)\right)=k_{s,i}m\frac{p(t)^2}{K_{i,1}K_{i,2}+K_2p(t)+p(t)^2}. \right. \tag{SI7}$$

The index i of the constants means that their values are individual for each protein, $k_{i,s}$ - initiation rate constant.

Taking into account that synthesis of a protein i takes certain time τ_I, assume that, at the current time, free concentration (r_i) of the protein i is proportional to the rate of the protein synthesis initiation

$$r_i(t)=k_i\frac{p(t-\tau_i)^2}{K_{i,1}K_{i,2}+K_2p(t-\tau_i)+p(t-\tau_i)^2}, k_i=\frac{k_{i,s}m}{k_{i,d}}. \tag{SI8}$$

To obtain the synthesis rate of an n-dimensional multimer, assume that its concentration (U) is proportional to the product, considering the monomers stoichiometry and concentrations of all free fractions of r_i proteins composing the multimer

$$U(t)=\frac{1}{K_U}\prod_i\left(k_i\frac{p(t-\tau_i)^2}{K_{i,1}K_{i,2}+K_2p(t-\tau_i)+p(t-\tau_i)^2}\right). \tag{SI9}$$

Hence the synthesis rates of multimers is

$$V_U(t)=\frac{k_U}{K_U}\prod_i\left(k_i\frac{p(t-\tau_i)^2}{K_{i,1}K_{i,2}+K_2p(t-\tau_i)+p(t-\tau_i)^2}\right). \tag{SI10}$$

In particular, neglecting the differences in the values of the same parameter depending on the type of a protein and assuming $\alpha=\frac{k_U}{K_U}$, the synthesis rate of the generalized ribosomal unit is

$$f=\alpha\left(\frac{\left(\frac{p(t-\tau_p)}{K_I}\right)^2}{1+\frac{p(t-\tau_p)}{K_I}+\left(\frac{p(t-\tau_p)}{K_I}\right)^2}\right)^{h_I} \tag{SI11}$$

Consider that the rate of ribosome degradation is proportional to the concentration of proteasomes. As a result, we obtain the following equation for the degradation parameter

$$g=\beta_1+\beta_2\left(\frac{\left(\frac{p(t-\tau_D)}{K_D}\right)^2}{1+\frac{p(t-\tau_D)}{K_D}+\left(\frac{p(t-\tau_D)}{K_D}\right)^2}\right)^{h_D} \tag{SI12}$$

SI 5. Analysis of the dynamic properties of the model (SI1), (SI11), (SI12).

The aim of this study is identical to the objectives of the study conducted in the Supplementary section SI2: identification of the chaotic dynamics in the expanded model for different parameter values. Based on the same reasons, the analysis was conducted for selective parameters. Just as before, equal values of parameters $\tau_1 = \tau_2 = \tau$, and integer values of the Hill coefficients (h_I, h_D) were used in the analysis.

Bifurcation diagrams calculated for 13 variants of parameter values depending on the value of the delay parameter τ illustrate the obtained results (Figure SI5.1). The dimensionless parameter values were converted to the dimensional units and the results are summarized in the Table SI6.1.

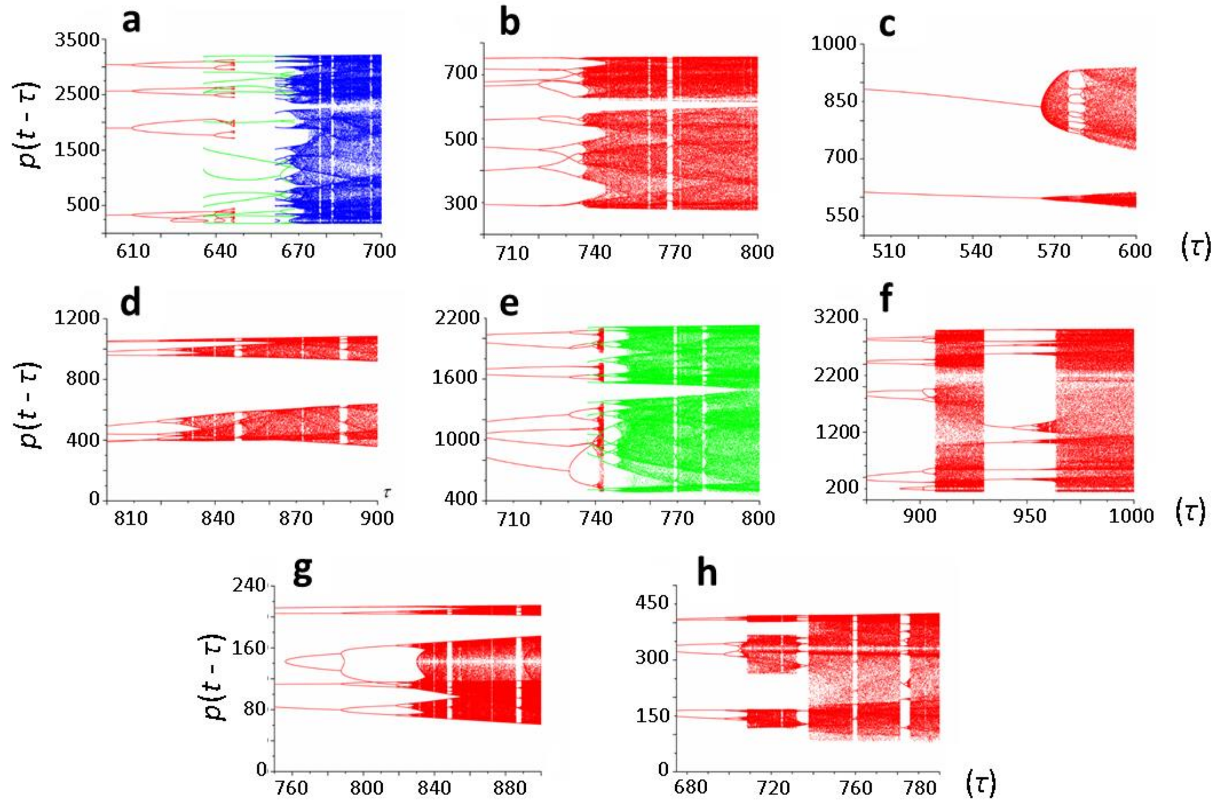

Figure SI5.1: Chaotic dynamics in the model of ribosome biogenesis for different parameter values. Bifurcation diagrams constructed from the intersection points of the equation (SI1), (SI11), (SI12) solutions with the Poincaré plane $p(t)$. (a) $p(t)$ = 2500, parameter values α = 20, K_I = 500, K_D = 5000, h_D = 3; (b) $p(t)$ = 500, α = 10, K_I = 500, K_D = 1000, h_D = 3; (c) $p(t)$ = 500, α = 10, K_I = 500, K_D = 1000, h_D = 4; (d) $p(t)$ = 2500, α = 12, K_I = 300, K_D = 3000, h_D = 2; (e) $p(t)$ = 2500, α = 12, K_I = 300, K_D = 3000, h_D = 3; (f) $p(t)$ = 2500, α = 12, K_I = 300, K_D = 3000, h_D = 4; (g) $p(t)$ = 300, α = 2, K_I = 50, K_D = 500, h_D = 2; (h) $p(t)$ =500, α = 2, K_I = 50, K_D = 5000, h_I = 2, h_D = 4. Parameter values h_I, β_1 and β_2, unless otherwise indicated, are equal to 1, 0.001 and 1, respectively.

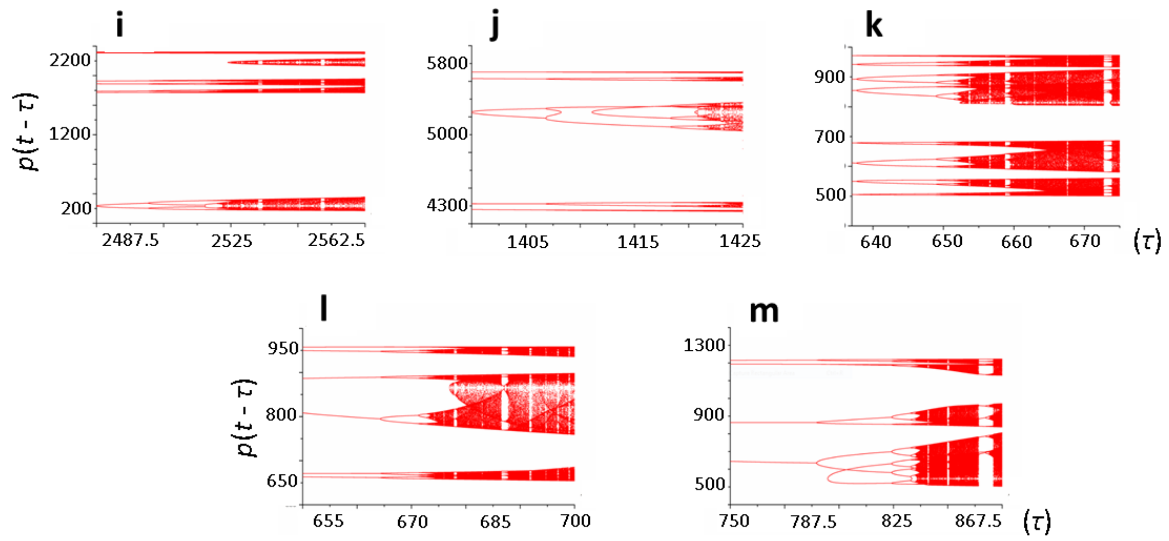

Figure SI5.1 (continued): Bifurcation diagrams constructed from the intersection points of the equation (SI1), (SI11), (SI12) solutions with the Poincaré plane $p(t)$. (i) Poincaré plane $p(t)$=500, parameter values $\alpha = 5$, $K_I = 50$, $K_D = 500$, $h_I = 10$, $h_D = 20$; (j) $p(t) = 2500$, $\alpha = 12$, $K_I = 50$, $K_D = 500$, $h_I = 30$, $h_D = 60$; (k) $p(t)$=500, $\alpha = 10$, $K_I = 50$, $K_D = 2000$, $h_I = 8$, $h_D = 2$; (l) $p(t)$ =500, $\alpha = 10$, $K_I = 50$, $K_D = 1000$, $h_I = 16$, $h_D = 4$; (m) $p(t)$=1000, $\alpha = 10$, $K_I = 50$, $K_D = 1000$, $h_I = 24$, $h_D = 6$. Parameter values β_1 and β_2 in all cases are equal to 0.001 and 1, respectively.

SI 6. Parameter values for which the chaos is observed in the model of ribosome biogenesis

Parameter values for which the chaos is observed in the model of ribosome biogenesis are summarized in the Table SI6.1. Algorithm described in the Supplementary section SI3 was used for converting the dimensionless parameters of the model to dimensional units.

Table SI6.1. Compliance between the dimensionless parameter values and the dimensional values at which the chaos is observed in the model of ribosome biogenesis

№*	Dimensionless/dimensional values of the model parameters (dimension)						function values	
	K_I (mcM)	K_D (mcM)	α (mcM/h)	τ (h)	h_I	h_D	$p(t)$	$g(t)$
1	10000/97.241	5000/48.620	100/32.347	488.2/3.176	1	5	1645.40	0.015
2	500/5.570	5000/55.704	20/6.672	524/3.787	1	4	1436.171	0.029
3	1000/10.209	5000/51.047	20/6.570	510/3.430	1	5	1567.191	0.016
4	500/7.019	5000/70.192	20/5.688	370/3.952	2	3	1139.725	0.025
5	500/23.369	1000/46.738	20/16.399	295/3.639	2	4	342.334	0.029
6	500/3.497	4000/27.979	20/4.715	668/4.715	1	3	2287.402	0.016
7	500/14.438	1000/28.876	10/14.309	737/3.219	1	3	554.096	0.010
8	500/14.438	1000/28.876	10/14.309	581/2.515	1	4	748.152	0.010
9	300/4.342	3000/43.418	12/4.426	828/7.031	1	2	1105.540	0.020
10	300/2.928	3000/29.282	12/4.180	741/4.493	1	3	1639.233	0.014
11	300/2.248	3000/22.477	12/3.709	908/4.763	1	4	2135.500	0.012
12	50/4.346	500/43.459	2/7.032	828/4.429	1	2	184.083	0.012
13	50/2.429	500/24.290	2/7.144	707/2.081	2	4	329.349	0.007
14	50/0.612	500/6.124	5/6.235	2522/5.361	10	20	1306.424	0.005
15	50/0.198	500/1.982	12/9.995	1422/1.465	30	60	4035.517	0.002
16	50/1.178	2000/47.100	10/9.979	653/3.335	8	2	679.404	0.012
17	50/1.149	1000/22.988	10/22.537	673/1.486	16	4	696.025	0.005
18	50/0.804	1000/16.085	10/25.290	836/1.151	24	6	994.731	0.003

* – parameter values №1 correspond to the model (1) calculations, shown in Fig. 4 and 5 in the main text of the article; parameter values № 2-5 – to the model (SI1)-(SI3) calculations, shown in Fig. SI2.1(a-d); parameter values № 6-18 – to the model (SI1),(SI11),(SI12) calculations, shown in Fig.SI5.1(a-m).

Dimensionless values β_1 in the parameter sets 1-5 were equal to 0.002 (table SI.6); and in the parameter sets 6-18 - to 0.001 (table SI.6) and were corresponding to the dimensional values 0.005 h^{-1} and 0.002 h^{-1}.

Dimensionless value β_2 in all parameter sets, shown in table SI.6, was equal to 1 and was corresponding to 2.31 h^{-1}.

The lower bound of the estimation of the value of parameter τ corresponds to the half-life of ribosomal proteins of 2 h.

Analysis of the data is shown in the main text of the article.
